# Analgesic agents administered by ambulance personnel to mountain bikers and hikers on trails in Western Australia

**DOI:** 10.1016/j.heliyon.2023.e21717

**Published:** 2023-10-26

**Authors:** Paul J. Braybrook, Hideo Tohira, Deon Brink, Judith Finn, Peter L. Buzzacott

**Affiliations:** a Prehospital Resuscitation and Emergency Care Research Unit (PRECRU), Curtin School of Nursing, Curtin University, Perth, Western Australia, Australia; b St John Western Australia, Belmont, Western Australia, Australia; c Discipline of Emergency Medicine, Medical School, The University of Western Australia, Perth, Western Australia, Australia; d School of Public Health and Preventive Medicine, Monash University, Melbourne, Victoria, Australia

**Keywords:** Emergency, EMS, Fentanyl, Injury, Methoxyflurane, Pain

## Abstract

**Objective:**

To describe the types of analgesic medications administered to patients who were attended by ambulance on recreational trails while mountain biking or hiking and report on the reduction in pain by these agents.

**Methods:**

This is a retrospective cohort study of patients attended by ambulance (2015–2021) after mountain biking or hiking, on Western Australia (WA) trails. All data were extracted from electronic patient care records created by ambulance personnel who attended the patient. We compared patient and case characteristics between mountain bikers and hikers and the reduction in pain scores achieved by different analgesics.

**Results:**

A total of 717 patients were included. Paramedics reported traumatic aetiology for mountain bikers in 92 % of cases and hikers in 58 % of cases. A pain score out of 10 was recorded for 538 (75 %) patients. The median (inter-quartile range) initial pain score was 6 (2–8) and the median final pain score was 3 (1–5). Around 48 % of these 538 patients reported ≥25 % reduction in their pain score. A reduction of ≥25 % in their pain score was greatest in those patients who received intravenous fentanyl (81 %), followed by patients administered multiple analgesics (72 %) and methoxyflurane only (52 %). Even 37 % of 134 patients who received no analgesia still reported ≥25 % reduction in their pain score by hospital arrival.

**Conclusion:**

Trauma was the most common reason mountain bikers and hikers on trails called an ambulance and a large proportion of these patients were in pain on ambulance arrival. Further work assessing the effectiveness of safe, non-opioid analgesics, additional to methoxyflurane, is needed to ensure non-registered practitioners such as first aid providers and event medical teams can offer suitable safe analgesics to these patients. Additionally, among patients given no pharmacological analgesic agent, almost half still achieved a >25 % reduction in their pain scores which reiterates the importance of non-pharmacological pain reduction strategies.

## Teaser

A descriptive study of analgesic agents administered by ambulance personnel to mountain bikers and hikers on trails in Western Australia.

## 1 Introduction

Despite the multiple health, social and economic benefits of recreational trail use, it must be set in the context of acute injuries which can occur when undertaking these activities. Common acute traumatic injuries in mountain biking have been reported to be predominantly lacerations and abrasions with more serious injuries being mainly upper extremity fractures [[1], [2], [3]]. Participation in hiking has increased in Australia over the past twenty years and it is estimated that around 6.9 % of the population participate in hiking each year [4]. Of the studies that investigate injury trends within national parks and other recreational destination, the majority of non-fatal injuries to hikers appear to be soft tissue injuries and located on lower extremities [[5], [6], [7], [8]]. There is potential for these cohorts of people to experience traumatic injuries or health conditions in remote, hard to access locations with extended prehospital contact times. These injuries can result in pain for this cohort of patients which should be appropriately managed by the attending ambulance personnel.

Within Western Australia (WA), the ambulance service utilises four analgesics for the treatment of acute non-cardiac origin pain. The analgesics are (1) oral acetaminophen (Panadol, GSK, Abbotsford, Victoria), (2) inhalational methoxyflurane (Penthrox, Medical Developments International, Springvale, Victoria, Australia), (3) fentanyl in intravenous (IV) and intranasal (IN) preparations (Orion Laboratories, Balcatta, Western Australia, Australia) and (4) IV/intramuscular (IM) ketamine (Pfizer, Sydney, New South Wales) [9]. These analgesics are all available within metropolitan locations and rural locations where paramedics are employed. However, in smaller rural and remote locations, the ambulance service is staffed by volunteer emergency medical technicians (EMTs). Fentanyl and ketamine can be administered only at locations where ambulance paramedics are available, whereas methoxyflurane can be administered by all ambulance staff in any location within WA. Ketamine can only be administered after a minimum loading dose of fentanyl has proved to be ineffective. In most rural locations within WA, which are without registered paramedics, acetaminophen and methoxyflurane are available [9].

Treatment of pain in the prehospital setting is often reported as being suboptimal, despite similar analgesic agents to those used in a hospital setting being available [10]. Where pain is not treated adequately, it can lead to short-term effects, including undue suffering, tachycardia and hypertension [11], making patient assessment difficult and/or masking potentially serious injuries. In the longer term, under-treatment of acute pain has been suggested to enhance the likelihood of post-traumatic stress disorder (PTSD), post-traumatic stress symptoms (PTSS), persistent pain associated with the initial injury and lower effectiveness of analgesia in subsequent events [12,13].

A review of analgesics in mountain rescue situations, not specifically mountain bikers or hikers, recommended the use of a range of medications including acetaminophen, methoxyflurane, fentanyl and ketamine [14]. These recommended agents are currently all in use with the WA ambulance service. One study reported that methoxyflurane has shown to be effective in mountain bikers [15]. Work has shown fentanyl to be effective in patients injured while skiing [16], however no previous research has investigated other analgesics used in recreational trail users requiring emergency medical services (EMS) attendance.

The aim of this study was to describe the types of analgesic agents used amongst trail users transported by ambulance within WA and report the changes in pain scores after administration of these agents.

## 2 Methods

### 2.1 Participants

We conducted a retrospective cohort study of all people transported to hospital by ambulance due to a mountain biking or hiking injury/medical event within WA between January 1, 2015 and December 31, 2021.

Approval was obtained from the Curtin University human research ethics committee under approval numbers HR128/2013-63 and HR128/2013-73.

The study participants comprised people of any age who were attended by ambulance and transported to a hospital while mountain biking or hiking on a trail within WA between January 1st^,^ 2015 and December 31st^,^ 2021. Cases were identified using St John WA (SJWA) electronic Patient Care Records (ePCR) completed by the attending ambulance personnel at the time of the incident. These ePCR records are formed by a systematic collection of patient health data and administrative information of the incidents (e.g., time of call, dispatch code, and coordinates of the scene). Cases were identified through a four-stage process. Initially, a geographical delineation was applied between metropolitan Perth and surrounding areas. All cases outside the metropolitan area and those in Kalamunda, Mundaring and Gosnells, which are inside the metropolitan area but have many recreational mountain biking and hiking trails, were included. All mountain biking and hiking trails available from the Trails WA website were downloaded and overlayed onto the non-metropolitan area already delineated by local government boundaries [17]. Trails WA is an independent body with a publicly available database of trails for public use. These trails were then buffered by approximately 50 m to ensure any cases logged with inaccurate GPS coordinates were captured. Any cases falling within these buffers were then also added to the dataset. Finally, one of the authors (PJB) undertook a manual review of free-text case summaries in the ePCR on the extracted data in each record identified by the above search methods to ensure cases were either mountain biking or hiking related and on trails. All subsets of mountain biking were included in the dataset, including downhill, cross-country and enduro-style riding. All types of hiking on trails were also included in the dataset, including trail running, bush walking, overnight hiking, and day hikes. Both competitive and non-competitive riding and hiking were included.

### 2.2 Exclusion criteria

Cases were excluded if the mountain biking or hiking was not trail-related. Cases who did not call an ambulance during the event, such as after returning home, were also excluded from the dataset. Where cases were attended by multiple ambulances, only the primary ambulance data was used to avoid duplication of cases. Cases in metropolitan Perth, except those in Kalamunda, Mundaring and Gosnells, were excluded because walking trails within this region are within parks or along rivers and do not have the similar remote, complicated access problems for EMS as do trails in non-metropolitan areas. Patients who were attended by ambulance but not transported to hospital were also excluded. For safety reasons, all patients who receive any analgesia are required to be transported to hospital.

### 2.3 Analgesia

Acetaminophen is given orally in a 1000 mg dose every 4 h to a maximum of 4 doses in a 24-h period. Methoxyflurane is administered by pouring a 3 ml dose into a green inhaler fitted with a charcoal filter. A second dose of 3 ml is indicated if patients are still in pain after 15 min. Fentanyl is available in both IV (100 μg/ml) and IN (450 μg/1.5 ml) administration. IV fentanyl is administered according to weight with a loading dose of 1 μg/kg for patients under 70 years old and 0.5 μg/kg for patients over 70 years old. Follow-up doses of 25 μg are approved for all adult patients every 5 min to effect. Paediatric patients (aged <17 years) are given 1 μg/kg up to a maximum of 25 μg with follow-up doses of 1 μg/kg to a maximum of 25 μg. Doses of intranasal fentanyl are 15 μg and 180 μg and are on a weight-based approach. Paramedic crews may also utilize ketamine for traumatic pain but only where the initial minimum fentanyl dose is shown to be ineffective. Ketamine is given IM at a dose of 1 mg/kg to a maximum single dose of 100 mg and with follow-up doses of 0.5 mg/kg, or IV at a dose of 10–20 mg with subsequent doses of 1 mg every 5 min. Acetaminophen, methoxyflurane and fentanyl may be administered concurrently. In this study, “multiple analgesia” includes any combination of available analgesics. It should be noted that clinicians are also encouraged through clinical practice guidelines (CPGs) to utilize a range of non-pharmacological methods of analgesia such as splinting, positioning of injured limbs, immobilization, utilization of ice packs and distraction techniques [9].

Treating clinicians are comprised of four levels of skillset: ambulance volunteers, ambulance officers, paramedics, and critical care paramedics (CCPs). Acetaminophen and methoxyflurane are available and approved for administration by all levels of clinician, whereas fentanyl and ketamine are approved for administration by paramedics, ambulance officers (under paramedic supervision) and critical care paramedics only. The administration of all medications and the doses indicated are controlled by SJWA CPGs [9].

### 2.4 Variables

The following data were extracted from the ePCR data: date of the case, age, sex, activity being undertaken, prehospital care interval (time from ambulance dispatch to arrival at a hospital), patient contact interval (time from ambulance arrival at the scene to arrival at hospital), aetiology of the case, paramedic reported fracture or dislocation, pain scores and the analgesia administered. Methoxyflurane is stated to be effective for just 30 min [9] and, with a clinical practice guideline limit of two doses per patient, the theoretical maximum analgesia time is 60 min [9]. To account for the average time for paramedics to reach a patient once they arrived on the scene (20 min), the patient contact interval was categorized into 0–60 min, 61–120 min and greater than 121 min. This categorization was chosen to give an indication of how many patients were outside the maximum methoxyflurane effectiveness of 60 min. Pain scores are recorded by an attending clinician after asking the patient to score their current pain from 0 to 10, with 0 indicating “no pain” and 10 indicating “the worst pain ever”. As per the SJWA CPGs, pain scores of 0 indicate no pain, 1–4 were categorized as mild pain, 5–7 categorized as moderate pain and 8–10 categorized as severe pain. Paediatric patients were assessed using the Visual Analogue Score for Pain Intensity (VAS-PI), specifically utilizing the PAINLOG (Schlenker Enterprises, Ltd., Lombard, IL) device by asking patients to identify how much pain they were in from the descriptive facial pictures and the clinician recording the corresponding number which was blinded from the patient. The VAS-PI also ranges from 0 to 10. The verbal numeric rating scale (VNRS) and VAS-PI have been shown to correlate highly in providing an equal assessment of pain scores [18]. Pain scores were taken from the first reported pain score (initial pain score) and the pain score prior to hospital arrival (final pain score). The change in pain scores were calculated by subtracting the final pain score from the initial pain score. To calculate whether a patient achieved a meaningful reduction in their pain score, a proportional reduction in pain was chosen as opposed to an absolute reduction. Previous work has indicated that patients with higher initial pain scores require a greater absolute reduction to achieve the same relative relief [19]. A systematic review of 37 studies reported that the median minimum clinically important difference (MCID) was around 23 % [20]; hence, in this study we considered a conservative reduction value of ≥25 % from the initial pain score as meaningful pain reduction. The presence of a fracture or dislocation was identified by the trained treating ambulance personnel through palpation and inspection for any deformity, crepitus or other obvious signs of fracture or dislocation.

### 2.5 Data analysis

We compared patient age, sex, analgesic agents received, patient care intervals(the time ambulance crews are in contact with the patient), and presence of clinician-reported fracture or dislocation between mountain bikers and hikers. Continuous data were described with medians and interquartile range and compared using a Wilcoxon rank-sum test. Categorical data were described with count and percentage and compared using a chi-square test. We analyzed the data using Stata 17.0 (College Station, TX: StataCorp LLC).

## 3 Results

### 3.1 Patient and case characteristics

A total of 717 patients requested to be transported to hospital by ambulance whilst mountain biking (n = 399, 56 %) or hiking (n = 318, 44 %) on a trail. Mountain bikers were significantly younger (p < 0.001) [median age 38 years (IQR 24–49) than hikers 49 years (31–63) for hikers] and significantly (p < 0.001) more likely to be male(84 % for mountain bikers vs 39 % for hikers) (Table 1).

**Table 1:** Descriptive statistics of mountain bikers and hikers transported by ambulance.

variable	Mountain bikers N (%) N = 399	Hikers N (%) N = 318	*P*-value
Median age^a^ (iqr)	38 (24–49)	49 (31–63)	p < 0.001
Sex^a^			p < 0.001
Male	336 (84)	124 (39)	
Female	63 (26)	193 (61)	
Patient contact interval			p < 0.001
Median minutes (iqr)	58 (39–83)	72 (44–116)	
Pre-hospital interval			p < 0.001
Median minutes (iqr)	87 (64–114)	115 (80–182)	
Pre-hospital interval			p < 0.001
0–59 minutes	86 (22)	42 (13)	
60–119 minutes	235 (59)	125 (39)	
120+ minutes	78 (19)	151 (48)	
Initial pain score			p < 0.001
Nil	65 (16)	114 (36)	
Mild (1–4)	86 (22)	62 (19)	
Moderate (5–7)	116 (29)	64 (20)	
Severe (8-10)	132 (33)	78 (25)	
			
Aetiology			p < 0.001
Trauma	373 (93)	185 (58)	
Medical	26 (7)	133 (42)	
Reported fracture/dislocation			p = 0.009
Yes	129 (32)	70 (22)	
No	270 (68)	248 (78)	
Analgesic agent administered			p < 0.001
Nil analgesia	125 (24)	153 (40)	
Iv fentanyl	131(25)	49 (13)	
Inhalational methoxyflurane	174 (34)	110 (29)	
Oral acetaminophen	52 (10)	48 (12)	
Intranasal fentanyl	22 (4)	20 (5)	
Im/iv ketamine	15 (3)	4 (1)	

a 1 patient did not have sex recorded and 3 patients did not have age recorded.

### 3.2 Pain scores

Of all patients attended by ambulance a total of 538 (75 %) had pain scores recorded, 179 (25 %) patients had no pain scores recorded and 134 (19 %) patients had pain scores recorded but with no analgesia administered. The initial pain scores of mountain bikers and hikers were significantly different. A higher proportion of hikers reported no pain [114 patients (36 %)] when compared with mountain bikers [65 patients (16 %)] (Table 1). The proportion of patients reporting moderate or severe pain was higher in the mountain biking group [248 patients (62 %)] than in the hiking group [142 patients (45 %)] (Table 1). A significantly (p < 0.001) higher proportion of ambulance attendances to mountain bikers were for traumatic reasons, 373 patients (93 %), compared with hikers, 185 patients (58 %). Significantly (p = 0.009) more mountain bikers than hikers had a fracture reported by the attending clinician (129 patients (32 %) for mountain bikers vs 70 patients (22 %) for hikers) (Table 1).

### 3.3 Analgesic agents used

In total, 1557 separate doses of analgesia were given to 439 (61 %) patients. Of the patients given analgesia, inhalational Methoxyflurane was the most common analgesic given to patients (n = 284 patients, 65 %), followed by IV Fentanyl (180 patients, 41 %), acetaminophen (100 patients, 23 %), IN fentanyl (42 patients,10 %), and ketamine (19 patients, 4 %).

A total of 199 patients had fractures or dislocations recorded by the treating ambulance personnel. For patients with fractures and/or dislocation, the most common analgesic given was methoxyflurane in 137 (47 %) patients, followed by IV fentanyl in 71 (24 %) of patients. For 519 patients without a reported fracture, almost half of the patients received no analgesic agent (256 patient, 49 %) and 147 (24 %) received methoxyflurane (Table 2). Of the 199 patients who had a fracture reported 22 (11 %) did not receive any analgesia. Over half of all patients who did not have a fracture reported (n = 263, 51 %) still had analgesia administered. Multiple analgesic agents were given to 85 patients (60 %) who had a fracture reported and 76 patients (14 %) who did not have a fracture reported.

**Table 2:** Type of analgesics administered to patients with and without reported fractures.

Analgesic	Fracture reported, n (%)	No fracture reported, n (%)
Nil	22 (7)	256 (42)
Oral Acetaminophen	35 (12)	65 (11)
Inhaled Methoxyflurane	137 (47)	147 (24)
IN Fentanyl	16 (5)	26 (4)
IV Fentanyl	71 (24)	109 (18)
IV/IM Ketamine	13 (4)	6 (1)
Total	294	609

### 3.4 Changes in pain scores by type of activities and type of agents

**Fig. 1:**
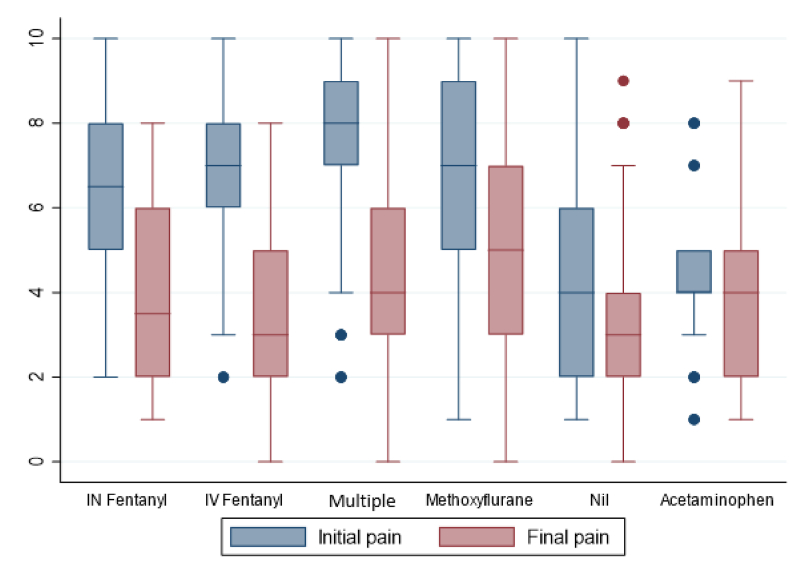
Initial and final pain scores (VNRS) for each analgesic agent.

**Fig. 2:**
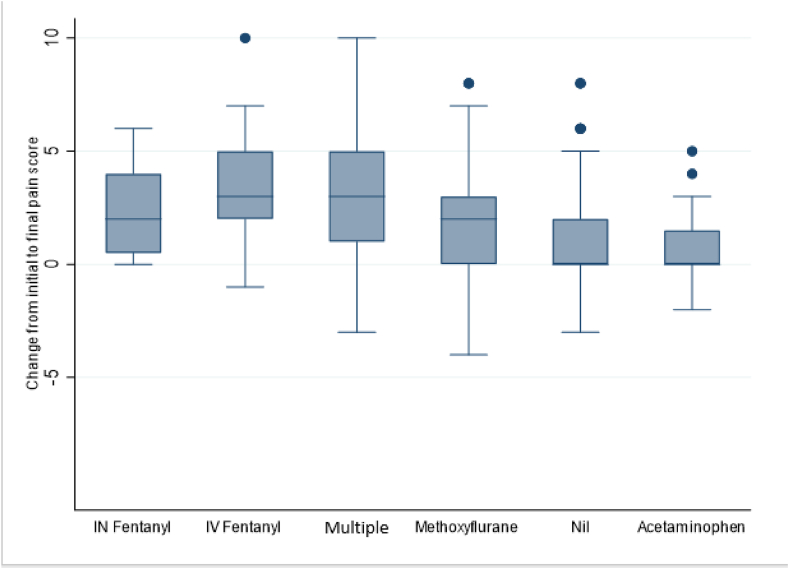
Overall decrease in patients pain score (VNRS) for each analgesic agent (negative value indicates pain scores reducing).

Overall, 536/717 (75 %) patients had a pain score recorded at the scene and another at hospital arrival. The median initial pain score was 6 (out of 10) (IQR 2–8) (Table 2) (Fig. 1). Of the 536 patients for whom pain scores were recorded both at the scene and at hospital arrival, 301 (57 %) achieved a meaningful pain reduction (Table 2). Initial pain scores were highest in the multiple analgesia group at 8 (7–9) followed by methoxyflurane at 7 (5–9), IV fentanyl at 7 (6–8) and IN fentanyl and 7 (5–8) (Fig. 1). Both acetaminophen and the nil analgesia group had similar initial pain scores at 4 (5–8) and 4 (2–6), respectively (Fig. 1). The change in pain scores achieved for each analgesic agent is shown in Fig. 2. Of the 536 patients reporting pain, 33 (6 %) recorded higher final pain scores than their initial score; and these are represented in Fig. 2 with negative pain score changes. Ketamine is not shown in Fig. 2 or 3 nor Table 2 due to low sample size with only two patients receiving ketamine alone and the remaining doses all being given as part of multiple analgesic treatments.

## 4 Discussion

We described the characteristics of a cohort of trails-based mountain bikers and hikers transported to hospital via ambulance focusing on the analgesics administered. In investigating prehospital analgesics used by the WA ambulance service we identified that for patients reporting pain either IV fentanyl or a combination of multiple analgesics achieved the highest proportion of minimum clinically important difference (MCID) pain reduction with 81 % and 72 % of patients reported a MCID in their pain score by arrival at hospital respectively (Table 2).

We found mountain bikers were more likely to be male and hikers female. This is supported by previous work which indicated that injured mountain bikers were approximately 70–92 % male [[21], [22], [23], [24], [25], [26], [27], [28], [29], [30], [31]]. There is limited research into medical events affecting low altitude hikers with most research being focused on Alpine regions. However, one study on aeromedical retrievals of hikers found between 43 and 53 % were female, which is consistent with our findings [32,33]. It is likely our results represent the sex bias associated with participation. Mountain bikers were significantly younger than hikers by over 10 years, 38 years compared with 49 years. Previous research has reported injured mountain bikers to be younger than we found, with average ages around 15–33, compared with our 38 [22,25,26,29,30]. Conversely, research has shown hikers requiring EMS assistance to be older than we found, with one large study reporting over 70 % of hikers being aged over 50 years old [34]. It is possible the differences in the median age of both cohorts in the present study is representative of the participation trends of both sports in WA.

Mountain bikers had significantly shorter prehospital intervals at 87 (64–114) minutes compared with 115 (80–182) minutes for hikers. To our knowledge no previous work has reported on the prehospital intervals of mountain bikers or hikers, so we are unable to make comparison with other regions. It is possible the difference between the prehospital interval in these cohorts is due to hiking trails being less centralized without direct access points and often being further away from metropolitan areas.

Over half of the patients (n = 390) described in this study reported moderate to severe initial pain and over 93 % of mountain bikers and 58 % of hikers called an ambulance due to trauma-related injuries. Whilst more mountain bikers than hikers reported medium to severe initial pain this is likely due to the greater proportion of call outs being for trauma related cases. More mountain bikers than hikers had the presence of a fracture reported by the attending clinician at 32 % compared with 22 %. Previous work found fractures constituted between 30 % and 43 % of reported injuries in mountain biking, consistent with our findings [26,29,30]. Whilst very little research has been completed on hiking specific injury epidemiology, some work has previously reported 9 % of EMS attended hikers to have a fracture, below our reported figure [32]. Mountain bikers had significantly more fractures than hikers with 32 % of all mountain biker cases sustaining a fracture compared to 22 % of hikers. However only 58 % of ambulance call outs for hikers were for trauma related causes compared to 93 % of mountain bikers. This makes the proportion of fractures from trauma related calls higher in hikers than mountain bikers. Despite the lower velocities and kinetic energy involved in these traumatic events to hikers it is possible this high proportion of fractures is due to the higher median age of hikers, a risk factor that has been previously linked with higher incidence of fracture [35].

Patients receiving multiple analgesics reported a slightly higher median initial pain score of 8 (IQR 7–9) when compared with IV fentanyl, with a median of 7 (IQR 6–8) and a slightly higher final pain score than IV fentanyl with 4 (3–6) compared to 3 (2–5). Previous findings of a large retrospective analysis comparing prehospital analgesic use in New South Wales, Australia found a multiple analgesic approach to be no different in effectiveness to fentanyl alone [36]. Similar work demonstrated the same to be true for paediatric patients with the effectiveness of a multiple analgesic approach being no different to fentanyl alone [37]. It is possible our multiple analgesic cohort showed slightly higher residual pain scores as it included patients from rural areas where only paracetamol and methoxyflurane were available as the multiple analgesic approach.

Patients who received only methoxyflurane reported a MCID in their pain 52 % of the time, meaning almost one in two patients did not receive a meaningful reduction in their pain score by hospital arrival. Patients given methoxyflurane had initial median pain scores of 7 (5–9), similar to both IV and IN fentanyl, but final pain scores for patients given methoxyflurane were higher than all other analgesics, with a median of 5 (3–7) compared with IV fentanyl with a median of 3 (2–5) or a multiple analgesic approach having a median pain score of 4 (3–6). This finding was congruent with a previous retrospective Australian prehospital study which found methoxyflurane to be effective in 59 % of the 19,235 of patients [36]. A prospective study into the use of methoxyflurane in mountain bikers with traumatic injuries found mean initial pain scores to be around 7, with mean pain score after 20 min to be around 4 [15]. It is likely our results are higher as the median patient contact intervals are greater than 20 min and the effectiveness of the methoxyflurane may have been decreased by the time the hospital arrival pain score was recorded. Previous work has shown fentanyl to be superior in patients reporting medium to severe pain when compared with methoxyflurane in both adult and paediatric patients [37]. Patients who were administered IV fentanyl alone achieved a MCID in their pain score 81 % of the time. As fentanyl is indicated for unlimited repeat doses if it is administered at appropriate time points, it is as reasonable to expect fentanyl to be as effective over 60 min as it is with cases lasting over 300 min. Patients who were administered methoxyflurane alone were likely to receive 60 min of analgesia at most, (the maximum effectiveness of two doses) which may explain the large difference in the proportion of patients having a MCID recorded for their pain. These results support previously reported findings that methoxyflurane is inferior to fentanyl in achieving a MCID in self-reported pain score [36,37].

Initial pain scores for the nil analgesia group were lower than those of the fentanyl, multiple analgesic and methoxyflurane groups at 4 (2–6). Patients who received no analgesia still achieved a MCID in their reported pain scores 49 % of the time, and these patients may have received pain relief from non-pharmacological means. Previous work investigating prehospital analgesia on paediatric patients found children whose pain was managed through non-pharmacological interventions did not differ in their pain score on ED arrival from children administered pharmacological analgesia [38]. The reasons for patients not being administered analgesia was not investigated by this study. Practitioners are instructed by CPGs to administer analgesia for patients reporting pain unless they refuse analgesia or have allergies to the available analgesic. As many rural locations have access to only methoxyflurane or acetaminophen, there is the potential that if a patient does not tolerate or have contraindications to methoxyflurane then there are limited pharmacological options for analgesia and, consequently, clinicians would have to rely on non-pharmacological means to reduce pain. These may include options such as distraction, splinting, ice packs and positioning, all of which are encouraged through the WA ambulance service CPGs. Clinicians did not routinely report non-pharmacological means of analgesia, thus it was not possible to investigate the frequency of their use. Of the 136 patients who reported pain but were not given pharmacological analgesia only 12 (9 %) had the presence of a fracture reported by the treating clinician (Table 3). This indicates that, where patients did have a fracture identified, they frequently received a form of analgesic agent.

**Table 3:** Pain reduction characteristics by analgesic agent.

	Nil analge-sia n = 134	Multiple analgesic n = 155	Methoxy-flurane n = 130	IV fentanyl n = 77	IN fentanyl n = 12	Acetam-inophen n = 28	All patients n = 536
Initial pain VNRS median (IQR)	4 (2–6)	8 (7–9)	7 (5–9)	7 (6–8)	7 (5–8)	4 (4–5)	6 (2–8)
Last pain VNRS median (IQR)	3 (2–4)	4 (3–6)	5 (3–7)	3 (2–5)	4 (2–6)	4 (2–5)	3 (1–5)
Median pain reduction % (IQR)	0 (0–43)	50 (20–63)	25 (0–50)	50 (29–63)	28 (10–62)	0 (0–43)	20 (0–50)
MPR achieved n (%)	49 (37)	112 (72)	67 (52)	62 (81)	6 (50)	10 (38)	308 (57)

IQR interquartile range, VNRS verbal numeric rating scale, MPR meaningful pain reduction.

For patients in this study who did have a fracture reported by a clinician, a multiple analgesic approach was the most common analgesic regime used (Table 3). Some patients treated in this study may have had methoxyflurane or acetaminophen alone due to being treated by event medics or other non-registered practitioners working in rural locations. One suitable alternative for these practitioners could be the use of a ketamine wafer. This is currently being trialled by SJWA in the form of Wafermine^TM^ (iX Biopjharma, Singapore) a sub-lingual 25 mg racemic ketamine formation and is being made available for ambulance personnel at certain locations where opioid analgesics are unavailable [9]. Considering the strong analgesic effects of ketamine, and a recently published review demonstrating it as non-inferior to fentanyl, this could prove to be a suitable alternative to methoxyflurane for prehospital patients with trauma [39]. Considering the high proportion of patients achieving a MCID in pain scores despite receiving no analgesia it is recommended that further work be undertaken into this cohort of patients to identify how this reduction in pain scores was achieved. If using non-pharmacological means such as distraction, splinting and positioning was effective then these techniques could be further developed for use in remote areas where limited pharmacological means are available. A further recommendation is that more research be carried out into the epidemiology of hiking-related injuries. Considering the low overall proportion of hiking ambulance call outs being for traumatic reasons but the high proportion of fractures reported at these call outs it would be beneficial to understand the nature of these fractures further to better inform EMS who are attending these patients in remote, resource limited environments.

### 4.1 Limitations

The limitations of this study include being retrospective and observational. A further limitation of this study was that 25 % of mountain bikers and hikers did not have pain scores recorded. Whilst some of these patients may not have been in any pain, a proportion were possibly missing due to noncompliance with ePCR data recording requirements, especially considering some were administered analgesics. A further potential limitation in the retrospective analysis of such a distinct cohort is the potential to miss cases for inclusion leading to a selection bias. To address this, a wide net was cast for initial potential case identification, followed by manually reviewing each case to confirm whether mountain biking or hiking related. The number of patients in the data set did limit the potential for sub-group analyses.

## 5 Conclusion

We found mountain bikers and hikers presenting with pain, predominantly from traumatic injuries, reported that fentanyl and multiple analgesics achieved a greater proportion of minimum clinically important difference in pain reduction compared with methoxyflurane or acetaminophen alone. Considering that, of all patients given analgesia, around 50 % achieved a meaningful reduction in their pain scores the analgesics currently in use by the ambulance service may not be appropriate for this cohort of patients. Further research is needed into providing suitable analgesics for this cohort of patients, especially in rural areas where limited medications are available.

## Data availability

Data is unavailable for public release due to patient confidentiality.

## CRediT authorship contribution statement

**Paul J. Braybrook:** Writing – review & editing, Writing – original draft, Validation, Methodology, Investigation, Formal analysis, Data curation, Conceptualization. **Hideo Tohira:** Writing – review & editing, Supervision, Methodology, Data curation. **Deon Brink:** Writing – review & editing, Supervision. **Judith Finn:** Writing – review & editing, Supervision, Project administration. **Peter L. Buzzacott:** Writing – review & editing, Supervision, Project administration.

## Declaration of competing interest

The authors declare that they have no known competing financial interests or personal relationships that could have appeared to influence the work reported in this paper.
